# Natural Language Processing Algorithm Used for Staging Pulmonary Oncology from Free-Text Radiological Reports: “Including PET-CT and Validation Towards Clinical Use”

**DOI:** 10.1007/s10278-023-00913-x

**Published:** 2024-01-12

**Authors:** J. Martijn Nobel, Sander Puts, Jasenko Krdzalic, Karen M. L. Zegers, Marc B. I. Lobbes, Simon G. F. Robben, André L. A. J. Dekker

**Affiliations:** 1 Department of Radiology and Nuclear Medicine, Maastricht University Medical Center+, Postbox 5800, 6202 AZ Maastricht, Netherlands; 2 School of Health Professions Education, Maastricht University, Maastricht, Netherlands; 3 Department of Radiation Oncology (MAASTRO), Maastricht, Netherlands; 4 GROW School for Oncology and Reproduction, Maastricht University, Maastricht, Netherlands; 5 Zuyderland Medical Center, Department of Medical Imaging, Sittard-Geleen, Netherlands

**Keywords:** Radiology, Reporting, Natural language processing, Free text, Classification system, Machine learning

## Abstract

Natural language processing (NLP) can be used to process and structure free text, such as (free text) radiological reports. In radiology, it is important that reports are complete and accurate for clinical staging of, for instance, pulmonary oncology. A computed tomography (CT) or positron emission tomography (PET)-CT scan is of great importance in tumor staging, and NLP may be of additional value to the radiological report when used in the staging process as it may be able to extract the T and N stage of the 8th tumor–node–metastasis (TNM) classification system. The purpose of this study is to evaluate a new TN algorithm (TN-PET-CT) by adding a layer of metabolic activity to an already existing rule-based NLP algorithm (TN-CT). This new TN-PET-CT algorithm is capable of staging chest CT examinations as well as PET-CT scans. The study design made it possible to perform a subgroup analysis to test the external validation of the prior TN-CT algorithm. For information extraction and matching, pyContextNLP, SpaCy, and regular expressions were used. Overall TN accuracy score of the TN-PET-CT algorithm was 0.73 and 0.62 in the training and validation set (*N* = 63, *N* = 100). The external validation of the TN-CT classifier (*N* = 65) was 0.72. Overall, it is possible to adjust the TN-CT algorithm into a TN-PET-CT algorithm. However, outcomes highly depend on the accuracy of the report, the used vocabulary, and its context to express, for example, uncertainty. This is true for both the adjusted PET-CT algorithm and for the CT algorithm when applied in another hospital.

## Introduction

Accurate staging is important for optimal treatment of cancer patients. The tumor–node–metastasis (TNM) classification system is widely used for uniform staging of malignancies [1]. Diagnostic imaging, performed with computed tomography (CT) alone or combined with positron emission tomography (PET-CT), is, in addition to the clinical information, the most important source of information for staging pulmonary oncology. All tumor-specific information necessary for proper TNM staging should be present in the radiological report, as this functions as the communication method between the radiologist and the referring clinician [2–4]. However, despite mentioning tumor-specific items in the report, final TNM classification is often not present in the free-text report.

Natural language processing (NLP) is helpful in processing free text, such as the radiological report, to extract and structure its content [5, 6]. In oncology, NLP can be used for several purposes, such as case identification, determining outcomes, identification of critical findings, or staging [7]. In radiology, several different NLP methodologies have been described in literature, and this number has been growing over the years [5, 8, 9]. Also, in nuclear medicine, several studies have been published on NLP and oncologic imaging, such as finding bone metastasis in free-text bone scintigraphy reports [10]. Efforts to explore the potential of NLP in this field are illustrated by research published on finding pulmonary nodules and its characteristics in radiological reports [11] and finding a Lung-RADS classification out of structured reports used in pulmonary CT screening [12], as well as identifying staging characteristics about lung carcinoma in free-text radiology reports [13–15]. NLP is able to extract the TN stage of pulmonary oncology from the free-text radiological report of diagnostic staging CT scans by analyzing the text for this specific information and was shown by a rule-based TN-CT algorithm [13–15]. This research showed that it is possible to automatically extract T and N stage from free-text radiological reports with accuracy scores of 0.84–0.85. This algorithm may assist the radiologist in improving their report and may thereby support the clinical staging process. It can be applied during the reporting process or just before finishing the radiological report. Such a staging tool may be very helpful, as it is known that many differences between reports exist, for example, on the follow-up recommendations of pulmonary nodules [16].

However, the studies that developed TN-CT algorithms were only trained on diagnostic CT scans, but nowadays, diagnostic 18F-fluorodeoxyglucose (18F-FDG) PET-CT scans are increasingly used because they add metabolic information (the uptake of FDG by a tumor or lymph node) in addition to the anatomical information provided by the CT scan alone, as well as to find distant metastasis.

This specific metabolic information is important in the final TNM stage by upgrading or downgrading a specific stage according to the presence or absence of 18F-FDG avidity. This metabolic information is particularly important for determining the lymph node status, because an enlarged lymph node is not always pathological and a small lymph node is not always benign. As such, the information of the PET-CT does add information to the CT scan, sometimes leading to a different outcome if the CT scan information is overruled by the additional information of the PET-CT scan. To be able to address this information, an extra functionality layer to the existing rule-based TN-CT algorithm is required, so that both CT and PET-CT can be staged using such a new TN-PET-CT algorithm.

The aim of this paper is to expand the existing TN-CT algorithm into a TN-PET-CT algorithm used and compare this algorithm with the existing TN-CT algorithm. The research question is to find out whether it is possible to modify the TN-CT algorithm to also include PET-CT-specific metabolic information in order to get a full TN-PET-CT algorithm.

## Methods

### Corpus Description

The necessity to acquire (written) informed consent was waived by the local ethics committee. At the participating large secondary care center, a training and validation set was created to be used for the training and validation of the new TN-PET-CT algorithm. These sets consisted of a consecutive case mix of radiological reports of diagnostic CT and PET-CT scans performed for primary staging of lung carcinoma. For the training set and validation set, respectively, 63 (24 CT, 39 PET-CT) and 100 (41 CT, 59 PET-CT) reports were consecutively included in the period from January to June 2020. The numbers of included scans for each set were based on the numbers used in a different study using the TN-*CT* algorithm in which the goal was 100 cases for the validation set [13]. For external validation of the TN-CT algorithm, all 65 chest CT scan reports were used as the external validation set.

The reports were constructed as free-text reports by a medical specialist, other than the authors, being a radiologist for the CT scans, or a nuclear medicine specialist for the PET-CT scans. These original reports were used in this study. The exclusion criteria were (1) restaging and follow-up reports, (2) cases with two primary tumors, and (3) incomplete reports. Because the TNM stage was not specifically mentioned in the radiological report, the reports where manually labeled with T stage and N stage according to the 8th TNM classification system by two authors (JMN, JK), with, respectively, 10 and 11 years of radiology experience. Before labeling, annotation guidelines were set to allow for consistent labeling (Appendix 1). In case of differences, consensus between the two authors was reached. Characteristics of the tumor stage of both groups can be found in Table 1.

**Table 1:** Cohort composition of training and validation set

	**Training *(n* = *63)***	**Validation *(n* = *100)***
*T1aN0*	1	2
*T1aN1*	0	0
*T1aN2*	0	0
*T1aN3*	0	0
*T1bN0*	4	6
*T1bN1*	0	1
*T1bN2*	1	0
*T1bN3*	0	0
*T1cN0*	3	6
*T1cN1*	1	1
*T1cN2*	1	2
*T1cN3*	1	0
*T2N0*	0	1
*T2N1*	0	0
*T2N2*	0	1
*T2N3*	2	0
*T2aN0*	4	4
*T2aN1*	0	3
*T2aN2*	3	3
*T2aN3*	3	5
*T2bN0*	2	2
*T2bN1*	0	0
*T2bN2*	1	1
*T2bN3*	2	5
*T3N0*	5	5
*T3N1*	1	2
*T3N2*	4	9
*T3N3*	3	4
*T4N0*	11	13
*T4N1*	2	0
*T4N2*	4	10
*T4N3*	4	14

The included reports consisted of different subheadings depending on whether the report was a specific CT or PET-CT based and included subheadings, such as *clinical details*, *description of the modality*, *used methods (including used agents)*, *different body parts*, and *impression.* Especially, the PET-CT scan report subheadings differed from the CT scans report, as a PET-CT examination is a whole-body examination, thus including subheadings of body parts other than the chest.

The training set was used for fine-tuning and finding all relevant concepts to the vocabulary used in the external institution to align the TN-*PET-CT* algorithm to the external vocabulary. In addition, this set was used to train the additional metabolic layer functionality of this new TN-*PET-CT* algorithm, before validation.

The external validation set was used to externally validate the TN-*CT* algorithm as this algorithm was constructed before in a different hospital [15], without any modifications.

### Algorithm Construction

#### TN-CT Algorithm: Determining T and N Stage on CT Scan Reports

**Fig. 1:**
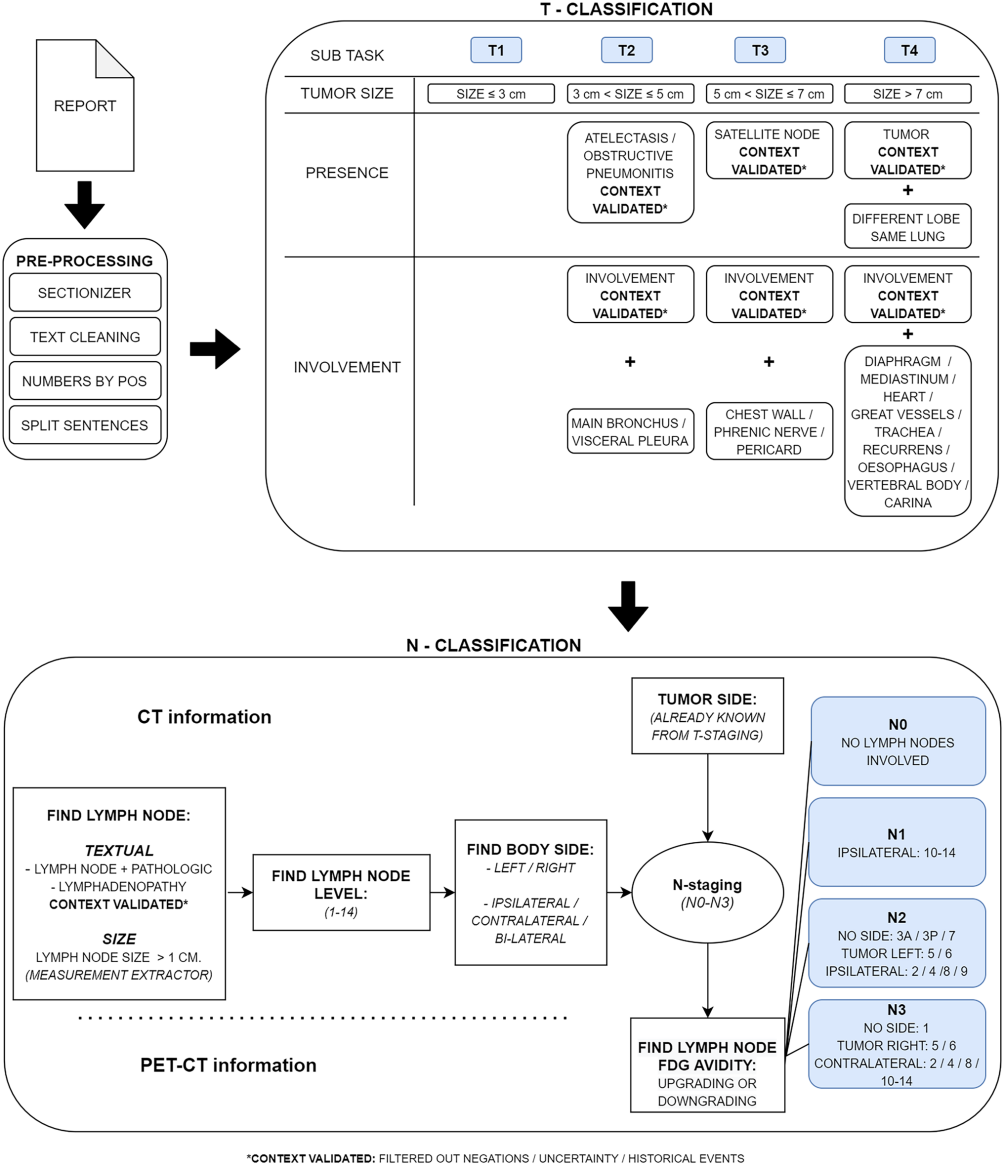
Schematic overview of the total TN staging process with its separate T and N classification and the new metabolic N stage layer to stage CT and PET-CT radiological reports at the bottom of the scheme

This study used the same rule-based staging algorithm for the T and N stage using Spacy and pycontextNLP as was used in prior studies [13–15], because both CT and PET-CT use the same characteristics and principles for staging pulmonary oncology. The approach is to extract the three important components for T staging, being tumor size, presence, and involvement, as well as finding all the lymph nodes, their size, lymph node level, and location side. After this, outcomes were matched, and final TN stage was assigned (Fig. 1). This process was performed by using regular expressions (RegEx), after the reports were pre-processed in which the sections were highlighted, the text cleaned, numbers extracted, and the sentences split.

#### TN-PET-CT Algorithm: Determining Additional Metabolic Information of PET-CT

To train and validate the new *TN-*PET-CT algorithm, an additional metabolic layer was newly built and added to the existing TN-*CT* algorithm to interpret the additional metabolic information according to ^18^F-FDG uptake by lymph nodes. This metabolic information of lymph nodes is of particular interest because an enlarged lymph node without FDG uptake is considered non-pathological, whereas a non-enlarged lymph node with FDG uptake is considered pathological. As such, the PET-CT specific information helps to fine-tune the final TN stage as it can upstage or downstage the N stage. The same fine-tuning step is possible for small pulmonary nodules which show FDG uptake and therefore are more suspect than pulmonary nodules lacking FDG avidity. However, this paper will only focus on lymph nodes, because for local staging of a lung tumor the PET-CT information about lymph nodes is most important.

To be able to add the metabolic information to the T and N stage functionality of the TN-*CT* algorithm, the reported FDG avidity with its accompanying concepts needs to be extracted and matched to the correct lymph node and the correct tumor side (Fig. 1). Because the lymph node and tumor side are already extracted by the TN-*CT* algorithm, only PET-specific vocabulary had to be trained and added to decide on its pathologic nature.

### Statistical Analysis

#### Training and Validation TN-PET-CT Algorithm

The training and validation set were compared, and separate T stage, N stage, and the combined TN accuracy scores were calculated. The precision, recall, and F_1_ measure were calculated to compare outcomes between the two datasets with the addition of the metabolic-specific layer used in this TN-*PET-CT* algorithm. Confusion matrices were built for the training and validation set of this new dataset to be able to compare the *actual TN stage* with the *predicted TN stage* of the new rule-based TN-*PET-CT* algorithm.

Error outcomes on data selection, context, concept matching, and reporter were analyzed and grouped by category to find difficulties still to overcome. A sub-analysis was made between the CT scan report group and the PET-CT scan report group of the training and validation set to separately compare the accuracy of the TN-*PET-CT* algorithm in these two groups.

#### External Validation TN-CT Algorithm

In the current study, the training and validation set used for building the new TN-PET-CT algorithm both consists of PET-CT and CT chest radiological reports reported in a large secondary care center. Because the TN-CT algorithm was developed in a different institution, this was the opportunity not to only expand it to an TN-PET-CT algorithm, but to also compare the performance of the prior TN-CT algorithm in the same clinical setting but at a different location. This external validation step is important to explore the functionality of the rule-based TN-CT algorithm, as it was only trained and validated in one medical institution on chest CT only [13]. For this reason, the CT reports of both the training and validation set were pooled and used for this external validation. The T stage, N stage, and the combined TN stage accuracy scores of the external validation set, as derived from the TN-CT algorithm, were calculated and compared with prior findings.

## Results

### Training and Validation TN-PET-CT Algorithm

The accuracy of the new TN-*PET-CT* algorithm*,* capable of staging both CT and PET-CT scans, was, respectively, 0.73 (*n* = 63) and 0.62 (*n* = 100) for the training and validation sets (Table 2).

**Table 2:** TN stage algorithm accuracy outcomes for TN-PET-CT and TN-CT algorithm. Outcomes of the training and validation sets and the external validation results are shown

	**TN-*PET-CT* algorithm**		**TN-*CT* algorithm**
	**Training (*n* = 63)**	**Validation (*n* = 100)**	**External validation (n = 65)**
**Accuracy T stage**	0.83	0.79	0.80
**Accuracy N stage**	0.86	0.80	0.89
**Accuracy TN stage**	0.73	0.62	0.72
**Accuracy T stage *(size only)***	0.75	0.71	0.69

The new TN-PET-CT algorithm was tested on the reports of both CT and PET-CT scans, and subgroup analysis of the CT report group and PET-CT report group in the training and validation set is shown in Table 3. The TN accuracy scores were 0.75 (*n* = 24) and 0.71 (*n* = 41) for CT in training and validation set and 0.72 (*n* = 39) and 0.56 (*n* = 59) for PET-CT (Table 3).

**Table 3:** TN stage algorithm accuracy outcomes for the separate CT scan reports and PET-CT scan reports using the TN-PET-CT algorithm for, respectively, the training and validation sets

	**TN-*PET-CT* algorithm**			
	**Training set** (*n* = 63)		**Validation set** (*n* = 100)	
	CT *(24)*	PET-CT *(39)*	CT *(41)*	PET-CT *(59)*
**Accuracy TN stage**	0.75	0.72	0.71	0.56

**Fig. 2:**
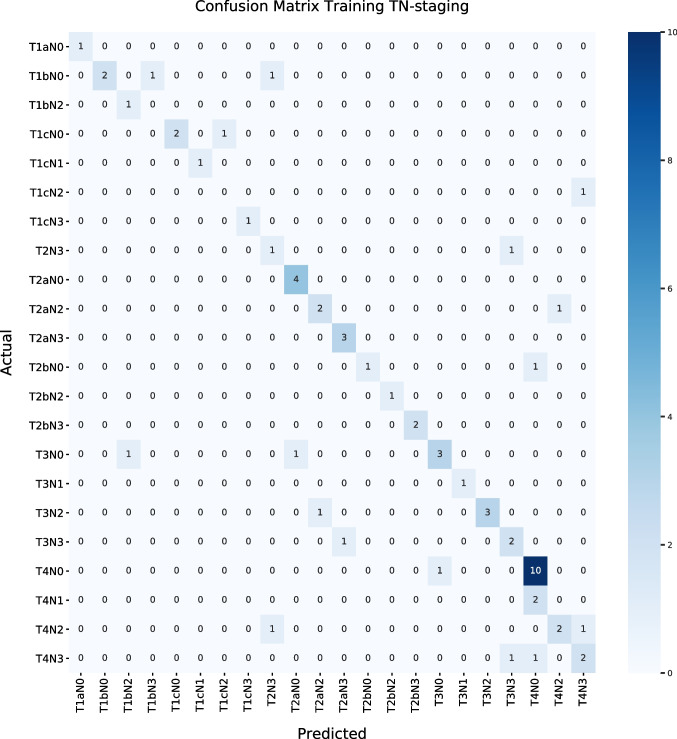

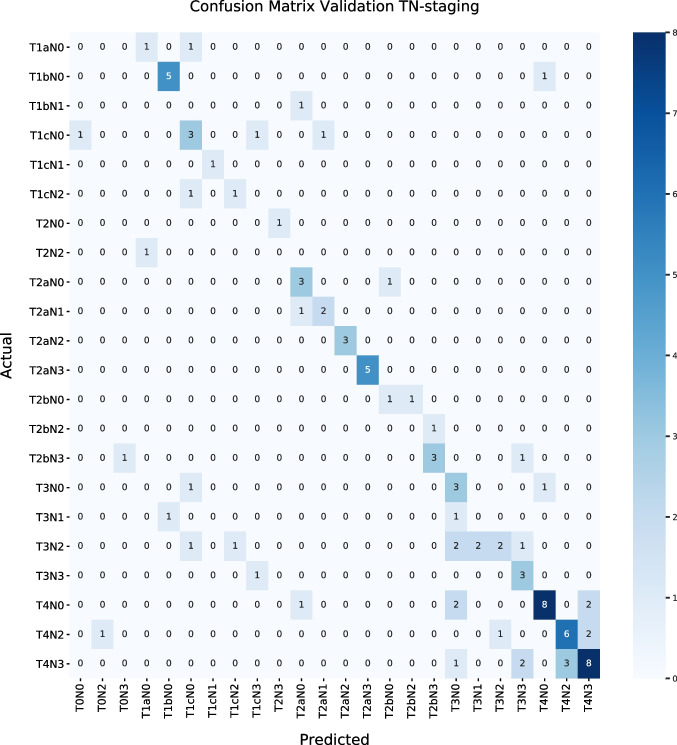
Confusion matrices of the TN-PET-CT classifier for the training (**a**) and validation (**b**) set

Precision, recall, and F_1_ measurement for the TN-*PET-CT* algorithm *and the* TN*-CT* algorithm can be found in Table 4. Confusion matrices of the training and validation set for the TN stage of the TN-*PET-CT* algorithm are shown in Fig. 2.

**Table 4:** Weighted precision, recall, and F_1_ scores of the TN stage for TN-PET-CT algorithm, as well as for the external validation set using the TN-CT algorithm *[12]

	**TN-*PET-CT* algorithm**			TN-*CT* algorithm*		
	Precision	Recall	F_1_ score	Precision	Recall	F_1_ score
**Training** *(overall)*	0.76	0.73	0.73			
**Validation** *(overall)*	0.68	0.62	0.63			
**External validation**				0.66	0.65	0.64

In Table 5, errors by category can be found that were made in the training and validation group. In total, 20 errors were made in the training set and 41 in the validation set, resulting in, respectively, 17 and 38 wrong classification scores.

**Table 5:** Errors per category by the TN-PET-CT algorithm

**Error group**	**Error type**	**Description**	**Training set (*n* = 63)**	**Validation set (*n* = 100)**
Data selection	Sectionizer	Subheadings not present or falsely not found — false correlation tumor or nodal description	2 *(2T)*	4 *(2T, 2N)*
	Measurement extraction	Size mismatch		2 *(1T, 1N)*
Context extraction	Missing	Context not matched because of missing/falsely matched modifier		3 *(2T, 1N)*
	Complexity	Context mismatch, wrong modifier detected: not or uncertainty	3 *(3N)*	3 *(3N)*
		PET or CT overruling	1 *(1N)*	3 *(3N)*
	Ambiguity	Matching not pulmonal lymph node	1 *(1N)*	2 *(2N)*
		Mentioning uncertainty	2 *(1T,1N)*	3 *(2T, 1N)*
Concept extraction	Missing	Synonym	2 *(2T)*	
	Ambiguity	Nodal description/station		
		Tumor-dependent atelectasis	1 *(1T)*	
		Abdominal mass		1 *(1N)*
	Complexity	Size description	1 *(1N)*	5 *(5T)*
		Invasion complexity	1 *(1T)*	
		Typing/speech error	2 *(2T)*	2 *(2N)*
		T3, implicit satellite node	1 *(1T)*	3 *(3T)*
		T4 multiple lobes, implicit mentioning	1 *(1T)*	2 *(2T)*
		PET, implicit mentioning		2 *(2T)*
Reporter	Wrong input	Incomplete node mentioning (location or pathological)	2 *(2N)*	3 *(3N)*
		No/incomplete size mentioned		3 *(2T, 1N)*
	Total errors		20*	41**

^*^20 errors in total, leading to 17 wrong classification scores

^**^41 errors in total, leading to 38 wrong classification scores

The accuracy score of the TN-CT algorithm on the external validation set was 0.72. Outcomes per subcategory, precision, recall, and F1 measurements can be found in Tables 3 and 4. Prior training and validation scores were 0.83 (*n*=47), and 0.87 (*n*=100) [13].

## Discussion

Free-text chest CT and PET-CT radiological reports can be used for the classification of pulmonary oncology according to the 8th TNM classification system [1]. The aim of this paper was focused on the process of constructing, training, and validating a rule-based NLP TN-*CT algorithm*, which is also capable to extract metabolic information from PET-CT scans, to also be able to use this new TN-*PET-CT* algorithm for staging PET-CT scan reports. Especially the information with regard to the metabolic activity of lymph nodes is of interest because the presence or absence of metabolic activity can change N classification. Because this study was performed in another institution than the institution where the TN-CT algorithm was originally developed, all 65 CT scan reports in the present study could be used for external validation.

The overall TN accuracy scores of the TN-*PET-CT* algorithm are 0.73 and 0.62 for the training and validation set. The accuracy scores of this new TN-*PET-CT* algorithm are lower than the scores of the TN-*CT* algorithm, which is the algorithm it is based on (0.84 and 0.85, respectively). Because the data sets used in building the TN-*CT* algorithm and the TN-*PET-CT* algorithm differ (only CT scan reports vs. CT + PET-CT scan reports), a full comparison of the outcomes is not possible. It does, however, point out a different functionality. Based on the 65 chest CT reports, the accuracy score of the TN-*CT* algorithm was 0.72 and comparable with the accuracy of the TN-*PET-CT* algorithm (0.75 and 0.71 in training set and validation set, respectively). Apparently, both algorithms perform similar for CT reports.

The addition of metabolic activity in the PET-CT reports did not improve the accuracy of the TN PET-CT algorithm in the training set (0.72), but in the validation set, the accuracy dropped to 0.56. Possible explanations for this low outcome can be the difference in cohort composition. First of all, the validation set entailed more T3 and T4 tumors compared to the training set (Table 1). As can be observed in the errors by category (Table 5), especially, T3 and T4 tumors were more difficult to match properly. This can be explained by the total number of items in the presence and involvement subtasks of the algorithm (Fig. 1), as there are more items in the T3 and T4 category that can overrule the tumor size subtask.

Secondly, a low metabolic information extraction functionality may be a factor. The effect can best be highlighted by the error category *PET or CT overruling* — in which the metabolic information of the PET-CT scan report resulted in a staging error of 1 (5%) in training and 3 in the validation set (7%) of the total errors made. This process of overruling is a difficult task because of difficulties in correctly extracting information of contradictory information about the same lymph node in one report. For instance, a lymph node can be reported as enlarged by its size but in the same sentence be reported as not pathologic due to the absence of FDG uptake resulting in the algorithm making the final — right — decision. Thirdly, the relatively small data sets (39 in training and 59 in validation set) may magnify small differences. For instance, there were relatively few overruling errors, but more in the validation set than in the training set, disadvantaging the validation set.

The comparison of the overall outcomes of the TN-*CT* algorithm with the TN-*PET-CT* algorithm show that all outcomes are lower in the new TN-*PET-CT* algorithm. Due to the small sample size, this outcome is not significant but probably highlights institution-specific difficulties, as the TN-*CT* algorithm was developed in an academic hospital, whereas the present study was conducted in a large secondary care center. Although errors in the external validation were not categorized, both in this study and the external validation, the way of reporting seems to be a strong influencing factor. For example, several reports were found that consisted of extensive prose, which makes it difficult for the algorithm to correctly match the context to the right concept. Furthermore, statements about the level of certainty [17] of a pathologic lymph node or the primary tumor seem to differ between institutions and between reporters. This decreases the algorithm’s performance because the algorithm is configured to only include pathology when it is stated as certain. Examples of different vocabulary use are words or phrases such as “suspected for,” “possible,” “probable,” or “suspicion of” but can also be highlighted by stating a differential diagnosis in which different pathologies are stated in the same sentence, rather than only stating the most likely diagnosis. As an example, a case with a tumor of 5 cm and clearly enlarged lymph nodes can be reported as “being suspicious for a lung carcinoma with lymph nodes suspect of lymphadenopathy,” while a different radiologist reports “a lung carcinoma with lymphadenopathy.” Interestingly, this difference is not caused by a difference in staging manner but rather in the way of stating the items found in the report. Of course, these items can be configured according to specific on-site preferences, but it is necessary that those items are reported always at the same manner and preferably in a standardized manner by every reporter.

The importance of standardized vocabulary, together with a clear report structure and more concordance to the TNM classification, is necessary to increase both report quality and staging accuracy by the algorithm. After all, all information necessary for staging should be stated in the text, and preferably the same way, so that it can be correctly recognized by the algorithm.

An important finding in Table 5 is the influence of human errors in many different error categories. For instance, in the error category *reporter* and *typing/speech error*, a final check by the reporter, just before finalizing the report, can prevent 4 (20%) and 8 (20%) of errors. In comparison, the mentioning uncertainty error group results in 2 (10%) and 3 (7%) of total errors, highlighting the importance of human influence in algorithm performance. Again, in clinical practice, these errors probably do not affect final staging, because — and in contrast to the algorithm — a human reader can understand these difficulties and can interpret implicit mentions.

Also, in the *data selection* category, several errors occur. This may be caused by a different format of the PET-CT report, including subheadings and specific vocabulary compared to the CT reports the algorithm was initially trained on. These differences can affect outcome.

However, throughout all categories, the biggest issue in this study seems to be the proper matching of context or modifiers to specific concepts. Of course, this can be caused by human errors, as already discussed, and especially when the report consists of prose, it is hard to accurately match items. But also without (direct) human influence, errors do occur. An example of such a mismatch can be tumor size extraction: because the algorithm is looking for the largest size in the report and anatomical matching to the right concept in a report with other sizes or numbers is a difficult task. For instance, a size of an enlarged adrenal gland, the diameter of a cystic kidney lesion or the thickness of a fluid collection can be mistaken for a size of a lung tumor. Especially in this study, this mismatching is of particular interest, because full-body PET-CT scans are included which increases the number of described organs and described (benign) entities in these organs with or without measurements, resulting in more mismatch possibilities compared to a single-chest CT scan report. A solution is a blacklist, as applied in this study, in which sizes can be excluded when not tumor or lymph node-specific. But this is not covering the entire spectrum yet, and additional rules need to be set making the algorithm more extensive. Moreover, adjusting or adding rules will most likely affect the effectiveness of other applied rules. Of course, machine learning can be helpful in differentiating these sizes or help with other matching problems, but large amounts of data are needed for this.

Furthermore, there might be reporting differences between institutions and between reporters, and the same seems to be true for reporting differences between the reporters that report a PET-CT and reporters that report a CT. In this study, the PET-CT scan reports were reported by the nuclear medicine specialist and the CT by a radiologist, both having a different vocabulary and reporting focus. After all, a radiologist mainly focuses on the anatomical landmarks, whereas a nuclear medicine specialist mainly focuses on the avidity and suspiciousness of the lesion(s) in the report.

These reporting differences probably do not lead to a different tumor stage in current clinical practice, as a human trained reader can match the report with the images of the (PET-)CT scan and can also interpret the used vocabulary in context to the overall findings of the report. However, for a rule-based NLP algorithm, it is difficult to grasp these different interpretation levels between and across different datasets as it — for now — lacks the possibility to check the images and the context of the overall report.

Overall, to allow for better accuracy of the algorithms, either better and clearer rules can be set when to call something a pathologic lesion, when to call something suspicious, or when to state that something is involved. ML can help to find a certain scale of uncertainty, but its use in clinical practice should be without too much variation to be consistent. Anyway, in both scenarios (clearer rules or ML), reporters should use some sort of standardization in stating uncertainty, with a possible alternative being emotion mining, in which reporter-specific certainty scale can be investigated.

### Limitations

One of the limitations of this study is that the datasets are probably too small, considering the low accuracy outcomes compared with the previous published data. Although the distribution between CT and PET-CT reports between both groups was the same and the included cases were a mix of consecutive cases, the validation set consisted of more higher staged tumors compared with the training and prior dataset. The cause of this difference is unclear but can be caused by the small numbers included.

A different limitation is that we are not able to calculate the interrater agreement between the two raters during this study, as it was done together in consensus. Although this was not the goal of this work, it can highlight the difficulties in extracting staging data. Finally, construction and validation of the metabolic layer were only performed in one single institution which can induce bias or overfitting. External validation of this TN-*PET-CT* algorithm is therefore still needed.

## Conclusion

It is possible to adjust the rule-based TN-*CT* algorithm to stage lung carcinoma according to the 8th TNM oncological classification system into a TN-*PET-CT* algorithm. However, the performance of this new TN-*PET-CT* algorithm is low, depending on several setting-specific factors, and the rule-based algorithm should be adjusted accordingly. When the rule-based approach is becoming the limiting factor, (specific) machine learning adjustments may be a promising tool.
